# Diagnostic of large vessel aneurysms as applicable to PPPM

**DOI:** 10.1186/1878-5085-5-S1-A86

**Published:** 2014-02-11

**Authors:** Dmitry I Cherepakhin, Yuri V Belov, Eduard R Charchyan, Olga M Bogopolskaya, Sergey V Suchkov, Igor A Yevtyushkin, Vladlen V Bazylev

**Affiliations:** 1 B.V. Petrovsky Russian Research Center for Surgery (RRCS), Moscow, Russia; 2 I.M. Sechenov First Moscow State Medical University, Moscow, Russia; 3 A.I. Evdokimov Moscow State Medical & Dental University (MSMDU), Moscow, Russia; 4 Federal Center for Cardiovascular Surgery, Penza, Russia

## 

Verification and confirmation of the diagnosis of cardiovascular disease is a very important segment of PPPM. Among PPPM-related clinical models, aortic aneurisms (AA) represent a niche illustrating great significance among cardiovascular diseases. Diagnosis and treatment of AA patients is appearing to be complicated because of cryptic clinical manifestations or masking other illnesses. There was a strong demand of finding a method of diagnostics which allows not only detecting of small vessel aneurism, but also its' progressing to large vessel aneurisms (LVA). We would suggest a multistep procedure to set up novel approaches for diagnosing early AA development. The assumption is based on molecular imbalance occurred in the tissue and bloodstream. Molecular changes are appearing to be important element making the diagnosis of LVA. And it’s thus important to stress the presence of key elements of the study, i.e., biopredictors or biomarkers, to create efficient diagnostic methods of LVA. The latter would allow diagnosing a multistep process of progressing aneurisms in record time and may help to reduce the disabling and mortality rates. Its major molecular biomarkers of LVA’s monitoring: Osteoprotegerin (OPG) has a high specificity in the AA analysis; it is an early marker for its diagnosis. The leading role in the pathogenesis belongs to metalloproteases (MMP-1, MMP-2), that destroy elastin in the aortic wall. The shift of balance between factors of activation and tissue inhibitors of MMPs enchases degradation of elastin and collagen, which leads to the AA development. One of the stages of MMPs activation is associated with the increase of factor TGF-B (transforming growth factor beta) and NFkB (nuclear factor kappa beta). An important role in the LVA development plays elastase and cathepsins, which damage the aortic wall without the help of MMP. But elastase is activated only when the level of cystatin C. Already at the aorta to the diameter of 5 mm clearly observed lack of cystatin C, which makes it an important marker of LVS’s diagnostic. The first step in modern diagnostic of LVA will become the screening of patients’ blood for detection MMP-2, MMP-9, OPG, cystatin C, TIMP-1, TIMP-2, IL-6, TNF-α, TGF-B and NFkB. In case of a positive result of serologic testing the presence of AA can be considered. The second step will be instrumental methods. It will be the imaging of the aorta - MRI or CT with contrast, which allows determining the localization of the AA. The third step will include the results of histodiagnosis and immunomorphology of biopsy specimens. This is algorithm will permit a detection of the disease at the *preclinical* stage, which allow to start timely pharmacoprevention. Findings research provide a way for designing primary preclinical diagnostic protocols for more adequate assessment of aortic wall status and early implementation of preventive drug therapy.

